# A novel transcription factor-like gene *SbSDR1* acts as a molecular switch and confers salt and osmotic endurance to transgenic tobacco

**DOI:** 10.1038/srep31686

**Published:** 2016-08-23

**Authors:** Vijay Kumar Singh, Avinash Mishra, Intesaful Haque, Bhavanath Jha

**Affiliations:** 1 Division of Marine Biotechnology and Ecology, CSIR-Central Salt and Marine Chemicals Research Institute, G. B. Marg, Bhavnagar (Gujarat), India

## Abstract

A salt- and drought-responsive novel gene *SbSDR1* is predominantly localised to the nucleus, up-regulated under abiotic stresses and is involved in the regulation of metabolic processes. *Sb*SDR1 showed DNA-binding activity to genomic DNA, microarray analysis revealed the upregulation of host stress-responsive genes and the results suggest that *Sb*SDR1 acts as a transcription factor. Overexpression of *SbSDR1* did not affect the growth and yield of transgenic plants in non-stress conditions. Moreover, the overexpression of *SbSDR1* stimulates the growth of plants and enhances their physiological status by modulating the physiology and inhibiting the accumulation of reactive oxygen species under salt and osmotic stress. Transgenic plants that overexpressed *SbSDR1* had a higher relative water content, membrane integrity and concentration of proline and total soluble sugars, whereas they showed less electrolyte leakage and lipid peroxidation than wild type plants under stress conditions. In field conditions, *SbSDR1* plants recovered from stress-induced injuries and could complete their life cycle. This study suggests that *SbSDR1* functions as a molecular switch and contributes to salt and osmotic tolerance at different growth stages. Overall, *SbSDR1* is a potential candidate to be used for engineering salt and drought tolerance in crops without adverse effects on growth and yield.

In nature, plants are often exposed to environmental stresses, which affect the bio-physiological activities of the plant. Salinity and drought stresses are major constraints for agricultural productivity worldwide^1^. Salinity costs agricultural crop production approximately $27 billion every year and therefore, particular attention is being focused on sustainable agriculture in salt-affected areas^2^,^3^. Salinity is predominant in arid or semi-arid areas, where water is limiting, and thus, agriculture is limited by drought and salinity under scarce rainfall^3^. In an economic cost model of salinity, it was estimated that farmers are often on the edge of profit or loss, and a small decrease in yield can have devastating economic consequences^3^.

The majority of agricultural crops grown nowadays are glycophytes (salt sensitive), and their productivity becomes commercially non-viable with an increase in salinity in the 4–8 dS/m range^4^, due to a decrease in yield of about 10%. Salt and drought stresses have similar effects on water potential, but salinity has additional cytotoxic effects. Salinity and drought both adversely affect photosynthesis, metabolic pathways and physiology. Consequently, this retards plant growth and can also lead to death^5^,^6^. Halophytes are naturally adapted plants that have the ability to complete their life cycle in a NaCl-rich environment; therefore, it is important to understand their abiotic stress tolerance mechanism. A signalling network is initiated under salt and drought stress conditions, which reprogrammes the expression of stress-responsive genes that are involved in different cellular, metabolic, biochemical and physiological processes^7^. Salt-tolerance genes from halophytes are considered potential key players with which to engineer plants to increase their salt tolerance^8^.

Abiotic stress causes a metabolic imbalance in cells, which promotes the production of reactive oxygen species (ROS) and results in damage to cell membranes, nucleic acids and cellular organelles, including the chloroplast^9^,^10^,^11^. Plants have developed a number of mechanisms to tolerate various stresses and stress-inducible genes play a key role in the regulation of stress tolerance and the maintenance of cellular homeostasis^12^,^13^. Previous studies have shown that the ectopic expression of abiotic stress-responsive gene(s) can positively regulate the stress tolerance of plants. However, bioengineering plants for stress tolerance requires knowledge concerning the key players of the stress-response network. However, it is difficult to distinguish critical genes that regulate the stress tolerance of the plant. Global transcript profiling has revealed that many genes are co-ordinately regulated by salt and drought stress^14^,^15^. A novel *MsZEP* gene was cloned from alfalfa for functional validation by heterologous expression in tobacco, and the gene confers drought and salt tolerance in transgenic plants^16^. The overexpression of a novel *Zmhdz10* in rice and *Arabidopsis* led to a significantly improved tolerance to oxidative stress caused by drought and salt stresses^17^. Similarly, the *IbZFP1* gene of sweet potato, which encodes a novel Cys2/His2 zinc finger protein, regulates ABA signalling, proline biosynthesis and ROS scavenging, and thus, improves salt and drought tolerance in transgenic *Arabidopsis*^18^.

Although several abiotic stress-responsive genes have been characterised, the quest for genes with novel functions continues, especially from halophytes. One extreme halophyte, *Salicornia brachiata*, which grows profusely on salt marshes, is a suitable candidate with which to study the salt stress-tolerance mechanism^19^. Several abiotic stress-responsive genes and promoters have been cloned from this halophyte to develop abiotic stress-tolerant transgenic plants^20^,^21^,^22^,^23^,^24^,^25^,^26^,^27^,^28^. *Salicornia* also possesses sulphur-rich seed-storage proteins^29^, unique oligosaccharides^30^ and metabolites^31^. The EST database of *S. brachiata* contains about 30% novel/unknown abiotic stress-responsive genes^32^ and we charactered a novel gene that is involved in salt and drought tolerance. A high transcript accumulation of the clone Sal-C-53.e1 (EB484704) was observed under salt and drought stress^32^, and was therefore selected for functional validation through a transgenic approach. In this study, we performed a detailed functional analysis of the *SbSDR1* gene by overexpressing it in tobacco plants to explore its roles in abiotic stress tolerance. The functional characterisation of this gene will reveal a novel regulatory molecular mechanism that can also be used to engineer crop plants that are tolerant to environmental stresses.

## Results

### A nuclear-localised gene *SbSDR1* differentially expressed under abiotic stress

The *SbSDR1* cDNA sequence was 728 bp (gene accession no. KF015229) long and consisted of a 5′-untranslated leader sequence (5′-UTR; 1–93 bp), an open reading frame (ORF; 94–483 bp), a 3′-UTR (483–705 bp) and a poly(A) tail of 23 base pairs (Figure S1). The 390-bp ORF encodes a peptide (accession no. AGU69247) of 129 amino acids with a molecular mass of 13.14 kD, a pI of 5.22 and an instability index of 36.93, which confirms that protein is stable in nature. The gene showed no sequence homology with sequences in the existing database, whereas the deduced peptide showed 68% sequence identity (with 98% query coverage) with an unknown/hypothetical or uncharacterised protein from *Beta vulgaris*. *In silico* analyses revealed that *Sb*SDR1 consists of helices and coils and contains an internal nuclear localisation signal (NLS; 41–53 amino-acid residues; IDKAKVAGAAEDV) without a cleavage site.

**Figure 1:**
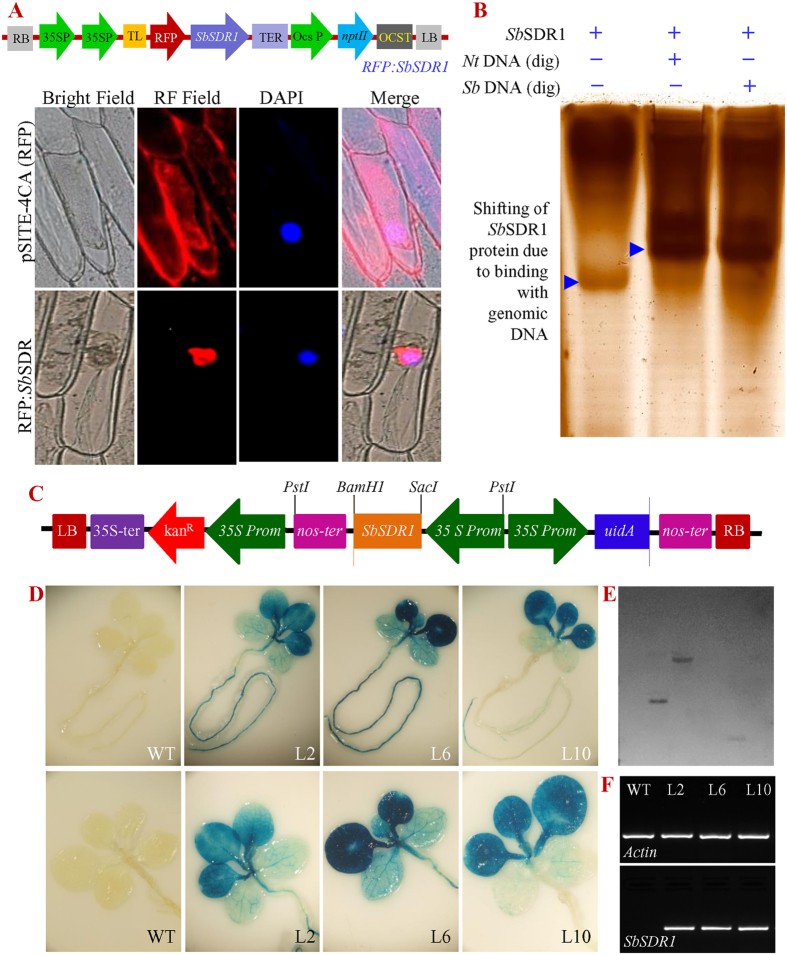
Sub-cellular localisation, DNA binding activity and confirmation of transgenic tobacco plants. **(A)** Gene construct *RFP:SbSDR1*, transient expression of RFP alone and RFP:*Sb*SDR1 translational fusion protein in transformed onion epidermal cells; **(B)** DNA binding activity of *SbSDR1* protein with genomic DNA of *Salicornia brachiata* and tobacco; **(C)** Schematic representation of *SbSDR1-pCAMBIA2301* plant transformation vector construct; **(D)** Histochemical GUS assay; **(E)** Southern hybridization to determine transgene copy number, and **(F)** Semi-quantitative RT-PCR of transgenic lines along with control plants.

Furthermore, transient expression of the recombinant RFP:*SbSDR1* protein confirmed that *Sb*SDR1 is a nuclear protein; in contrast, the evenly distributed expression of RFP alone was detected in the entire cell region in a subcellular localisation study (Fig. 1A). The *SbSDR1* ORF of 390 bp was amplified from cDNA and genomic DNA of *S. brachiata*. Comparative sequence and Southern blot analyses revealed a single exon structure and a single copy of the gene, respectively, and confirmed an intron-less genomic organisation of the gene (Figure S2).

Differential expression of *SbSDR1* was observed in *S. brachiata* under varying stress conditions; expression was up-regulated at each time-point compared to the control treatment (Figure S3). The expression level was rapidly induced by NaCl (two-fold at 6 h), then decreased after 12 h (compared to at 6 h) and again increased at 24 h. The expression of the gene increased concomitantly with increasing drought stress and reached a maximum (3.28-fold) at 24 h. Similarly, the transcript level increased gradually up to 12 h (4.03-fold) following heat stress and subsequently decreased slightly (2.78-fold). The transcript was also up-regulated (1.7- to 2.1-fold) by cold.

### The *Sb*SDR1 acts as transcription factor

*In silico* analysis predicted that the biological function of *Sb*SDR1 is to regulate metabolic processes and gene expression. Subcellular localisation analysis confirmed that the protein is localised to the nucleus. Furthermore, DNA binding property was assayed by a electrophoretic mobility shift assay. For this, *SbSDR1* protein was expressed in *E. coli* and the 13-kDa protein was extracted (Figure S4). The DNA/protein interaction showed a clear shift of the protein band, which confirmed the binding specificity of *Sb*SDR1 to genomic DNA (Fig. 1B).

### Molecular analyses of transgenic tobacco plants

Healthy leaves from T_1_ transgenic tobacco lines (T1 to T16 lines) overexpressing the *SbSDR1* gene (Fig. 1C), and wild type (WT) plants were harvested and screened for the presence of transgenes by PCR and histochemical GUS assays (Figure S5). The three lines L2, L6 and L10 that showed the highest *GUS* expression were selected (Fig. 1D) for further functional analysis using morphological, physio-biochemical and molecular analyses. Southern blot and semi-quantitative RT-PCR analysis confirmed the presence of a single copy of the transgene and a high expression of the *SbSDR1* gene, respectively in all selected lines (Fig. 1E,F).

### Ectopic expression of the *SbSDR1* gene improves plant growth under salt and osmotic stress

**Figure 2:**
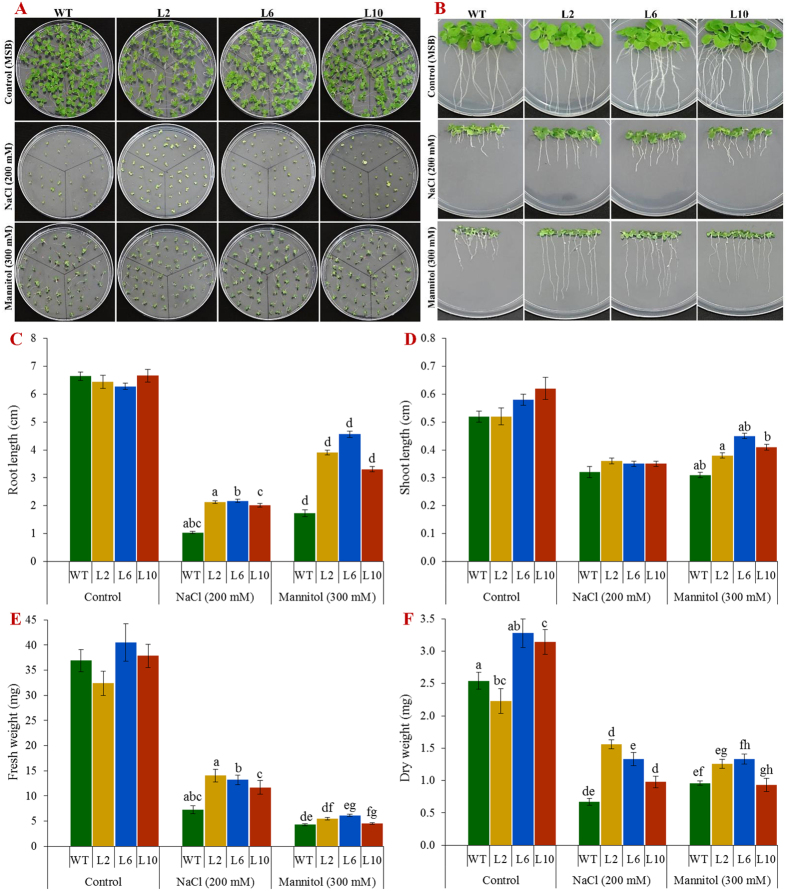
Plant growth analyses under salt and osmotic stress. **(A)** Seed germination of transgenic tobacco plants under different abiotic stress; **(B)** Comparative growth analysis of WT and *SbSDR1* plants under control and stress conditions. Comparative study of **(C)** root length; **(D)** shoot length; **(E)** fresh weight, and **(F)** dry weight of transgenic (L2, L6 and L10) and WT plants under salt and osmotic stress. Bars represent means ± SE and values with similar letters are significant at *P* < *0.05*.

Transgenic lines (L2, L6 and L10) showed a significantly (*p* < 0.05) higher percentage seed germination than control plants (WT) in stress conditions (Figs 2 and S6). The percentage seed germination of WT was about 58 and 82%, whereas that of the transgenic lines was 73–95 and 93–97% under salinity and osmotic stress, respectively. Although the growth of all plants under stress was lower than that of control plants, the reduction in growth was smaller in transgenic lines compared to in WT plants (Figs 2 and S7). Growth parameters, including fresh weight, dry weight, shoot and root length were significantly (*p* < 0.05) higher in transgenic lines (L2, L6 and L10) compared to those in WT plants under stress conditions (Fig. 2).

### Ectopic expression of the *SbSDR1* gene enhances physiological status of plant under stress

**Figure 3:**
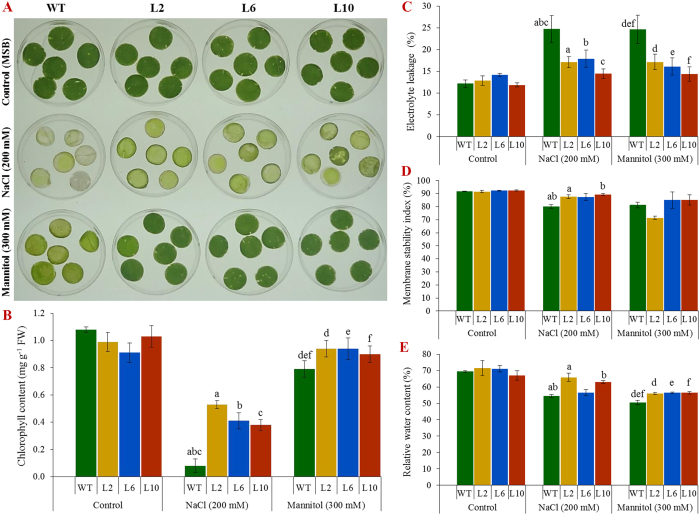
Leaf disc assay and physiological analyses of transgenic lines. **(A)** Leaf disc assay; **(B)** total chlorophyll content; **(C)** electrolyte leakage; **(D)** membrane stability index, and **(E)** relative water content from leaves of WT and transgenic (L2, L6 and L10) plant under control, salinity and osmotic stress conditions. Bars represent means ± SE and values with similar letters are significant at *P* < *0.05*.

**Figure 4:**
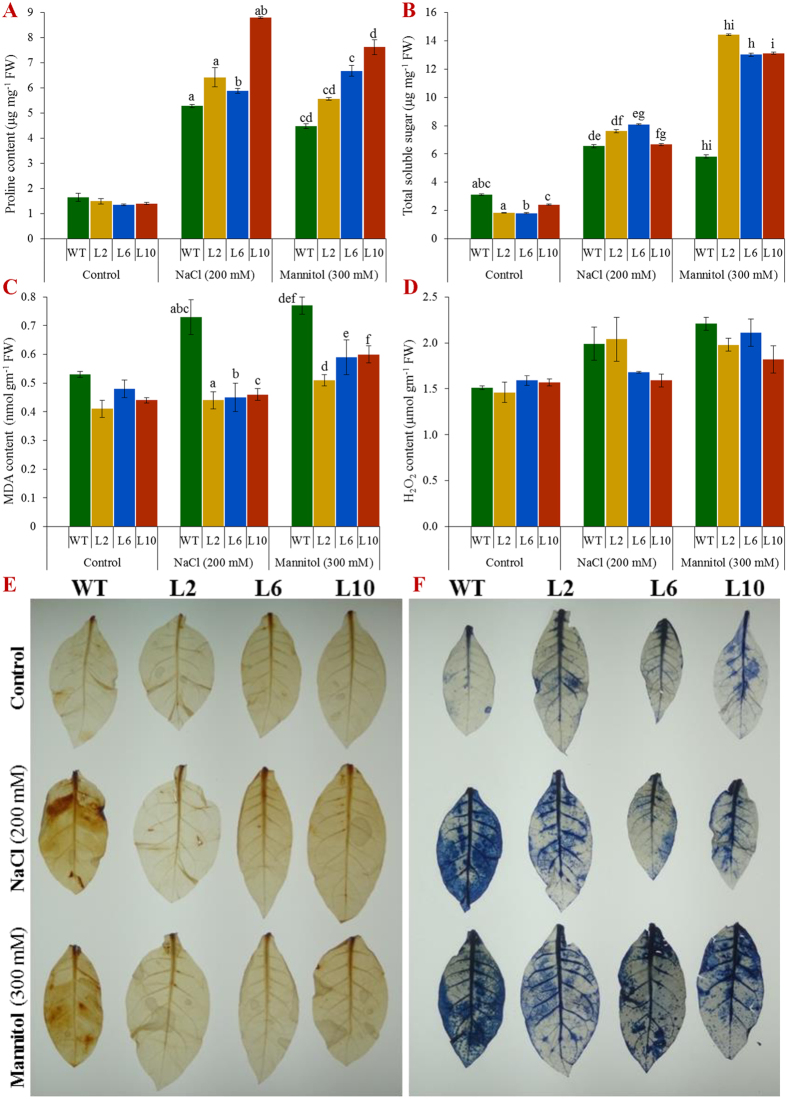
Biochemical analyses and *in-vivo* localisation of peroxide and superoxide free radicals of transgenic tobacco plants under abiotic stress. Estimation of **(A)** proline; **(B)** total soluble sugar; **(C)** MDA, and **(D)** H_2_O_2_ contents in WT and transgenic (L2, L6 and L10) plants under salinity and osmotic stress condition. *In-vivo* localisation of **(E)** peroxide by DAB, and **(F)** superoxide free radicals by NBT staining of transgenic (L2, L6 and L10) and WT leaves. Bars represent means ± SE and values with similar letters are significant at *P* < *0.05*.

Salt and osmotic stress induced less damage in transgenic lines compared to in control plants (Figs 3 and 4). Leaf segments of wild type plants showed a lower viability and senesced, whereas transgenic lines exhibited less necrosis under salt and osmotic stress (Fig. 3A). Furthermore, a reduction in cellular chlorophyll content was observed under stress conditions in all plants, but a significantly (*p* < 0.05) a higher chlorophyll content was observed in transgenic plants than in WT plants (Fig. 3B).

In control conditions, physiological parameters such as electrolyte leakage (EL), the membrane stability index (MSI), relative water content (RWC) and the concentration of proline and total soluble sugars were comparable in wild type and transgenic lines (Figs 3 and 4). Transgenic lines showed a significantly (*p* < 0.05) higher RWC and MSI, but a reduced EL under stress conditions compared to control plants (Fig. 3C–E). Furthermore, transgenic plants exhibited a considerably (*p* < 0.05) higher concentration of the osmoprotectants proline and total soluble sugars in transgenic lines compared to in wild type under salinity and osmotic stress (Fig. 4A,B). Thus, transgenic lines exhibited a better physiological status under stress conditions.

### The *SbSDR1* regulates ROS buildup under salinity and oxidative stress

The MDA and H_2_O_2_ contents were similar in all plants under control conditions, but showed a significantly (*p* < 0.05) lower accumulation in transgenic plants than in wild type under stress conditions (Fig. 4C,D). Furthermore, in an *in vivo* localisation study, WT plant leaves accumulated more O_2_^−^ and H_2_O_2_ than transgenic lines exposed to salt and osmotic stress (Fig. 4E,F).

### Ectopic expression of the *SbSDR1* gene provides salt and osmotic endurance

**Figure 5:**
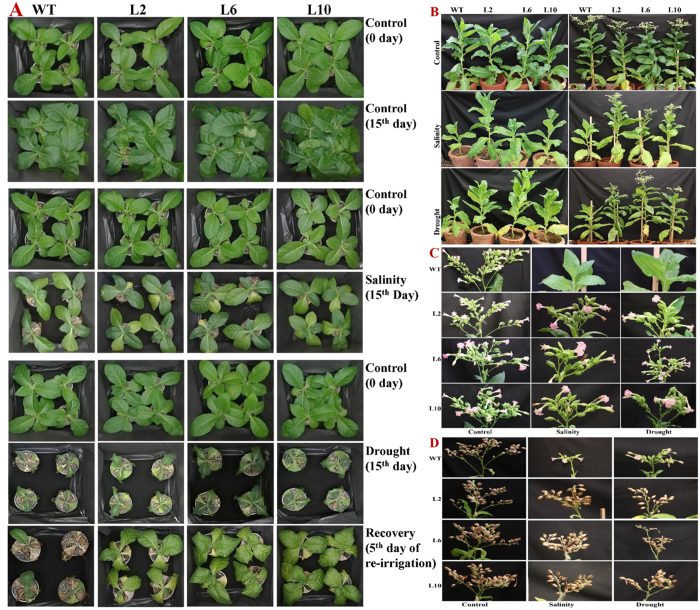
Comparative plant growth studies under salt and osmotic stress. **(A)** Morphological studies; **(B)** life cycle study; **(C)** flowering stage, and **(D)** mature (seed) stage of WT and *SbSDR1* transgenic plants at control and stress conditions. Plant images were documented as per stages of plants grown under control condition.

Thirty-day-old wild type and transgenic tobacco plants (L2, L6 and L10) with uniform growth were subjected to salt and drought stress in greenhouse conditions (Fig. 5A). The growth of all plants was comparable in control conditions, whereas that of WT plants was significantly affected by salt and drought stress. Although the growth of transgenic plants was suppressed, they showed better growth than WT. Furthermore, a higher degree of chlorosis was observed in wild type plants than in transgenic plants under salt stress. Retarded growth and wilting were observed in wild type plants compared to transgenic lines under drought stress and after re-irrigation, transgenic lines recovered rapidly compared to wild type plants.

**Figure 6:**
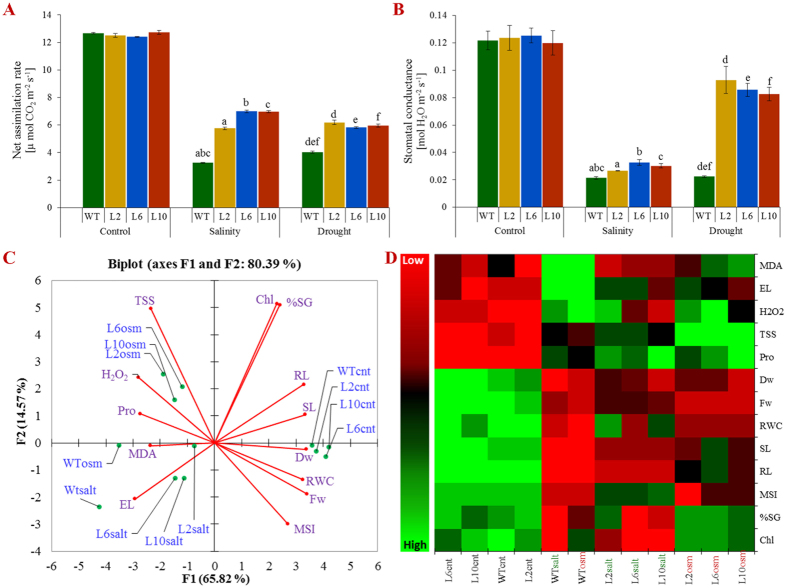
Physiological and multivariate data analyses of transgenic lines. **(A)** Net photosynthesis; **(B)** stomatal conductance; **(D)** an integrated comparative Bi-plot based principal component analysis with first two principal components, and **(E)** Heat map showing the differential response of transgenic lines (L2, L6 and L10) and WT plants under un-stress and stress (NaCl and osmotic) conditions. Bars represent means ± SE and values with similar letters are significant at *P* < *0.05*.

Similarly, the growth of WT plants in the field study was severely inhibited by salinity and drought treatment, whereas the growth of transgenic tobacco plants was less affected. Initially, all plants showed retarded growth, but subsequently, the growth of the transgenic lines gradually improved and the plants completed their life cycle under the stress treatment, whereas the WT plants did not (Fig. 5B). The transgenic plants were significantly taller and healthier than the WT plants and eventually flowered (Fig. 5C,D). In unstressed soil conditions, the number of inflorescences, flowers, pods and pod weight were the same in the transgenic and control plants (Figs 5 and S8). Furthermore, in the field, net photosynthesis and stomatal conductance were also similar under the normal (control) conditions for wild type and transgenic plants, but decreased with increasing salinity and drought stress (Fig. 6A,B). However, in comparison to WT, transgenic lines performed much better and showed a higher net photosynthesis and stomatal conductance.

### Differential morpho-physio-biochemical response of transgenic plants under stress condition

Principal component analysis (PCA) was used to distinguish the morphological, biochemical and physiological responses of transgenic and control plants under normal and various stress conditions (Fig. 6C,D). All plants (transgenic lines and control) showed comparable responses in the unstressed conditions, as revealed by the bi-plot analysis, in which transgenic and WT plants clustered together (cnt). Control plants showed similar responses under stress conditions, whereas transgenic lines exhibited a differential response, and clustered at an axis, and thus, revealed a similar response to environmental stress. The integrated heat-map shows the differential plant responses to the variables of the stress conditions. Individually, the morphological, physiological and biochemical responses of plants explained 96.15, 89.68 and 88.32% of the variation, respectively (data not shown), whereas together, they explained 80.39% of the variation, because of the varying response, as shown by the heat map.

### The *SbSDR1* gene involves in transcriptional regulation of host stress responsive genes and transcription factors

**Figure 7:**
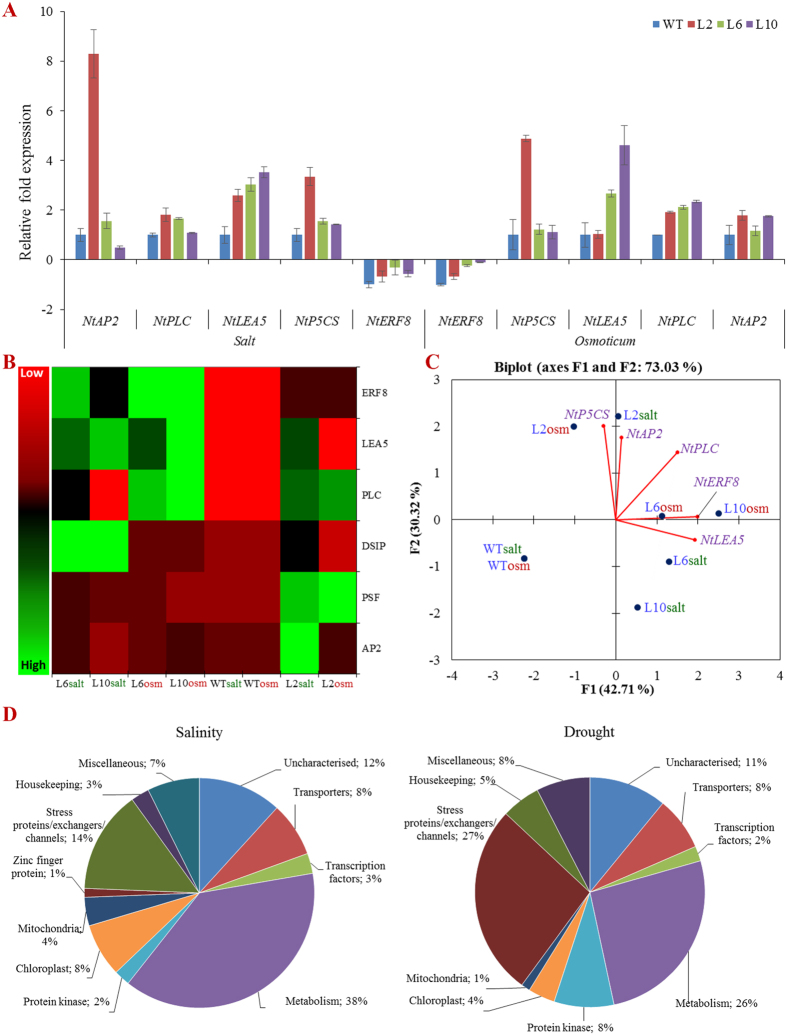
Transcript expression analysis and microarray-based functional classification of host stress responsive genes. Comparative **(A)** transcript expression profile; **(B)** Heat map, and **(C)** principal component (Bi-plot) analysis of *AP2* domain-containing transcription factor (*NtAP2*), phosphoinositide-specific phospholipase C (*NtPLC*), late embryogenesis abundant protein 5 (*NtLEA5*), delta 1-pyrroline-5-carboxylate synthetase (*NtP5CS*) and ethylene-responsive transcription factor 8 (*NtERF8*) genes under un-stress and stress (NaCl and osmotic) conditions. **(D)** Functional classification of differentially expressed genes of *SbSDR1* overexpressing transgenic tobacco plant under abiotic stress conditions. Genes differentially expressed in *SbSDR1* plant under stress condition were normalised with transcript of WT plants treated with same stress.

The expression of ten genes that encoded different transcription factors and other gene(s) of the host plant (*i.e.* tobacco) were studied. Five out of these ten genes were differentially expressed in transgenic plants under stress conditions (Fig. 7A). The expression of a gene encoding an *AP2* domain-containing transcription factor (*NtAP2*) was highly upregulated (eight-fold) under salt stress in transgenic plants. The phosphoinositide-specific phospholipase C (*NtPLC*), late embryogenesis abundant protein 5 (*NtLEA5*) and delta 1-pyrroline-5-carboxylate synthetase (*NtP5CS*) genes were upregulated in transgenic lines upon salinity and osmotic stress. In contrast, the gene encoding ethylene response factor 8 (*NtERF8*) was downregulated in transgenic lines, but was more down-regulated in WT plants in stress conditions. The heat map and PCA analysis further confirmed that genes encoding transcription factors were differentially expressed in stress conditions (Fig. 7B,C).

A whole-transcript expression array of tobacco, containing 272,410 EST gene-probe sets, was used to study the differential expression of host genes under high salinity and drought-stress conditions. A hierarchical cluster analysis and scattered plot confirmed the differential expression of host genes under abiotic stress conditions (Figures S9 and S10). A total of 12,490 and 5,142 genes showed at least a two-fold up- (>2) or down-(<−2) regulation under high salinity and drought stress, respectively, at *p* < 0.05 using ANOVA. In total, 1,998 genes were differentially expressed by more than eight-fold (<−8 or >8) under salinity stress, whereas 236 genes were differentially expressed more than five-fold (<−5 or >5) under drought stress (Figure S11). Out of the 1,198 genes with an eight-fold change in expression under high salinity, 1,169 genes were up-regulated, whereas 29 genes were down-regulated. Similarly, under drought stress, 167 genes were up-regulated, whereas the remaining 69 were down-regulated. In total, 31 genes were commonly expressed under salt and drought stress. The microarray analysis revealed the up- and down-regulation of many genes encoding transporters, ion exchangers, stress-related proteins, metabolic enzymes and transcription factors; some of the important genes/proteins are listed in Table 1. The differentially expressed genes/proteins were further categorised into different groups according to their biological functions (Fig. 7D). Approximately 38% and 26% genes/proteins that were differentially expressed under salt and drought stress, respectively, were associated with metabolism, whereas 14% and 27% of genes/proteins were stress-responsive or ion exchangers, respectively. Transporters represented 8% of all differentially expressed genes/proteins; however, about 12% of the genes/proteins were categorised as uncharacterised genes/proteins. About 2–3% of the transcription factors were upregulated under stress conditions.

## Discussion

Salinity is an emerging threat to agriculture and it has a detrimental effect on plant growth by imposing two simultaneous stresses; toxic salt ions and water stress. Although several abiotic stress-responsive genes have been characterised, research has focused on identifying novel gene functions for deciphering the salt stress tolerance mechanism and identifying candidate genes for developing resistant crops^8^,^33^. To explore potential novel genes for developing salinity tolerance in crops, here, we identify a salt- and drought-responsive gene, *SbSDR1*, from *Salicornia* and functionally characterised it in tobacco for the first time. The studied gene can be used to improve the salinity and drought tolerance of the agricultural crop.

No significant similarities were found between the nucleotide and deduced amino acid sequences of *SbSDR1* and other genes/proteins in a NCBI-BLAST sequence homology search; therefore, the gene is considered to be novel. The single copy of the intron-less *SbSDR1* gene (Figure S2) is differentially expressed under different environmental stresses (Figure S3). The expression of *SbSDR1* was elevated under salt (two-fold at 6 h), drought (3.3-fold at 24 h), heat (four-fold at 12 h) and cold (two-fold at 24 h) stress. Similarly, abiotic genes, such as *SbpAPX*, *SbGSTU*, *SbMT*-2 and *SbUSP* from *S. brachiata* were differentially expressed under different stress conditions^5^,^20^,^22^,^28^.

Sub-cellular localisation experiments confirmed that *Sb*SDR1 is a nuclear protein (Fig. 1A) and DNA/protein interactions revealed the binding specificity of *Sb*SDR1 to genomic DNA (Fig. 1B). *In silico* analyses also predicted that the *SbSDR1* protein is nuclear-localised and potentially regulates metabolic process and gene expression. The *SbSDR1* protein does not have a fixed conformation, because of the high content of disorder-promoting amino-acid residues (A, E, G, K, P, Q and S) and therefore, is considered to belong to the family of intrinsically disordered proteins (IDPs). Extensive post-translational modifications of IDPs have been reported and *Sb*SDR1 has 14 phosphorylation sites, which potentially facilitate protein–protein interactions^34^. Plant dehydrins, which are also IDPs, interact with ROS to protect cellular proteins from oxidative damage under stress conditions^35^. As an IDP, *Sb*SDR1 is protected during stress conditions and might transcriptionally regulate genes^26^. The *SbSDR1* protein also possesses structural flexibility and thus, can easily modulate biological functions by regulating metabolic processes and gene expression. The ability of *Sb*SDR1 to bind genomic DNA of *Salicornia* and tobacco confirmed that it functions as a transcription factor. The *in vitro* binding of *Sb*SDR1 enables it to regulate a large number of stress-responsive genes by binding to their regulatory regions. *In silico* analysis, subcellular localisation and the binding of *Sb*SDR1 to the genomic DNA of *Salicornia* and tobacco genomic DNA suggest that *Sb*SDR1 might functions as a transcription factor. A novel salt-induced gene, *LcSAIN1*, cloned from Sheepgrass (*Leymus chinensis*), conferred salt stress tolerance by activating the expression of transcription factors and functional genes in transgenic plants under salt stress^33^. Similarly, *Sb*ASR-1 protects the plant from salinity and drought stress and functions as a transcription factor in transgenic groundnut^26^.

Out of sixteen transgenic tobacco lines, three lines – L2, L6 and L10 – showed a high level of *GUS* expression (Figs 1D and S5) and, therefore, were selected to understand further the role of *SbSDR1* in abiotic stress tolerance. A single gene integration, a high expression of the transgene and a high seed germination percentage (Figs 1E, 2A and S6) were observed in the selected lines under stress conditions. Furthermore, the transgenic lines showed enhanced plant growth, including fresh weight, dry weight and shoot and root length under different stress conditions, compared to the control plants (Figs 2 and S7). The enhanced plant growth in the transgenic lines demonstrates that overexpression of *SbSDR1* reduces the adverse effects of stress. The degree of injuries caused by stress in a plant are commonly studied by assessing the physiological status, by measuring EL, the MSI, RWC, lipid peroxidation (MDA content), and the concentration of proline and total soluble sugars^12^,^13^. Notably, the ectopic expression of *SbSDR1* in tobacco promotes salt and osmotic tolerance by modulating the physiology of plants, including RWC, EL, MSI, proline, total sugar and MDA (Figs 3 and 4). A significant increase in RWC, MSI, proline and total soluble sugars alleviates stress injury, and thus, stress-induced damage is reduced in transgenic lines compared to WT, whereas lipid peroxidation and EL, which are common stress markers, decreased in transgenic lines. Proline is considered to be an osmolyte, a ROS scavenger and also a molecular chaperone that protects the cell from stress-induced damage^12^. Cells undergo metabolic rearrangements and activate regulatory networks in response to different environmental stresses, including salinity and drought^12^,^13^.

It is well established that plants often accumulate ROS under abiotic stress conditions, which leads to oxidative stress^9^. A reduction in the total chlorophyll content, which is a marker of cellular stress was observed in stress conditions, due to ROS generation in the chloroplasts^36^. Transgenic plants showed a significantly higher chlorophyll content and reduced leaf senescence and H_2_O_2_ content under oxidative stress (Figs 3 and 4). In this study, it was observed that *SbSDR1* inhibits ROS accumulation under salinity and oxidative stress (Fig. 4). Abiotic stress also leads to the generation of superoxide radicals^10^,^11^. The results confirmed that control plants (WT) accumulate more MDA, O_2_^−^ and H_2_O_2_ than transgenic plants and thus, confirm the role of *SbSR1* in contributing to oxidative stress tolerance by regulating ROS scavenging activities. Other abiotic stress-responsive genes, such as *SbpAPX1*, *SbNHX1*, *SbUSP* and *SbASR-1* from *S. brachiata*, contribute towards ROS homeostasis under salt and osmotic stress condition^5^,^6^,^25^,^26^.

Abiotic stress tolerance, including salt and drought tolerance, depends on the growth stage and is regulated at different developmental stages. Because the stress tolerance at one stage of plant growth does not correlate with tolerance at other stages^5^. Therefore, the performance of *SbSDR1*-overexpressing tobacco lines was assessed at different stages and in field conditions (Figs 5 and 6). The growth of WT plants was severely affected in comparison to that of transgenic tobacco lines, which were unaffected. Tobacco plants that overexpressed *SbSDR1* were significantly taller and healthier than WT plants, which showed retarded growth, wilting and chlorosis symptoms. Typical flowering and seed-setting were observed in transgenic lines but not in WT. In fact, the *SbSDR1* overexpressing plants performed better in the field and completed their life cycle in time under abiotic stress conditions, whereas the WT plants did not (Figs 5, 6 and S8). This study demonstrates that the ectopic expression of *SbSDR1* confers salt and osmotic tolerance at different developmental stages of the plant in pots and also in the field. Many studies have demonstrated that different salt-responsive genes, such as *SbNHX1*, *SbpAPX1* and *SbASR-1*, improved the tolerance of crops such as jatropha, castor and peanut to oxidative stress^24^,^25^,^26^,^37^. The *SbpAPX* gene confers salinity and drought tolerance to tobacco plants at different developmental stages^5^. Similarly, the overexpression of the novel gene(s) *LcSAIN1, MsZEP* and the TF, *Zmhdz10* in plants leads to the improved tolerance to oxidative stress that is caused by drought and salt stresses^16^,^17^,^33^.

Quantitative real-time PCR confirmed that the *SbSDR1* gene is involved in the transcriptional regulation of host stress-responsive genes and transcription factors, such as *NtAP2, NtPLC, NtLEA, NtP5CS* and *NtERF8* under stress conditions (Fig. 7A). Microarray analysis demonstrated the differential expression of several stress-responsive genes and transcription factors in *SbSDR1* transgenic plants compared to in WT plants under salt and osmotic stress (Table 1 and Fig. 7D). In this study, genes that were differentially expressed in transgenic plants under stress conditions were normalised using transcripts of WT plants treated with the same stress, to rule out the possibility that genes in WT tobacco plants are differentially expressed due to stress. Microarray analysis revealed the upregulation of genes encoding transporters, stress-related proteins, signalling components and transcription factors, as well as uncharacterised proteins. Salinity and drought stresses have a strong impact on gene expression, and many genes and stress-associated transcription factors associated with cellular metabolism are differentially expressed, to modulate the physiology of the plant to combat stress-induced injuries^7^,^12^,^13^. Previous microarray studies demonstrated that the overexpression of genes such as *OsACA6* and *OsCPK4* leads to the up-regulation of stress responsive genes/proteins, oxidative-burst proteins and transcription factors in transgenic rice^38^,^39^. The functional categorisation and distribution of differentially expressed genes in transgenic *SbSDR1* tobacco plants revealed that the majority of these genes encode proteins/enzymes involved in the maintenance of cellular metabolism^39^. Microarray results also suggest that *GmDREB1* activates the expression of many soybean-specific stress-responsive genes under different abiotic stress conditions^40^. Similarly, the overexpression of *DPB3–1* enhanced heat-stress tolerance via unknown transcription factors without growth retardation and yield reduction in rice^41^. In conclusion, the up-regulation of transcription factors, salt-responsive channels, transporters and enzymes/proteins involved in cellular metabolism in transgenic plants confirms that *SbSDR1* functions as a molecular switch and thus, confers abiotic stress tolerance to crops (Figure S12).

## Conclusion

In this study, a novel gene, *SbSDR1*, was functionally characterised. The overexpression of *SbSDR1* resulted in enhanced tolerance against salinity and drought stress. Morphological, biochemical, physiological and molecular evidence confirmed that transgenic plants are more tolerant to salinity and drought stress than wild type. Furthermore, the results provide direct evidence that *SbSDR1* contributes to tolerance to salinity and drought stress without yield loss by regulating stress-responsive genes. The functional characterisation of a novel abiotic stress-responsive gene provides new insights into the bio-physiological responses of plants at the molecular level to salt and drought stresses. The results further support the potential of *SbSDR1* as a candidate gene for the genetic engineering of crop plants to enhance tolerance to salt and drought stress.

## Methods

### Cloning, bioinformatics and transcript profiling

The Sal-C-53.e1 gene clone (EB484704), showing no significant homology/identity with existing database (NCBI/EMBL) and exhibited high expression under salt and drought stress, therefore, selected for the study^32^. Primers were designed from partial EST gene sequence of Sal-C-53.e1 gene clone (EB484704), a gene of unknown function (designated as *Salicornia brachiata* salt & drought responsive 1, *SbSDR1*) was made full length using RACE (rapid amplification of cDNA ends), cloned and sequenced (Table S1). The *SbSDR1* gene sequences were analysed for homology and conserved motifs. Amino acid sequences, deduced from nucleotide sequences, were characterised *in silico* using Expert Protein Analysis System^42^ (ExPASy). For expression analysis, one-month-old *S. brachiata* seedlings were transferred to a hydroponic system (½ MS with 8/16 h dark/light cycle at 25 °C) for 15 days. Acclimatised plants were subjected to different abiotic stresses such as salinity (250 mM NaCl), desiccation, heat (45 °C) and cold (4 °C). Total RNA was isolated from each treated plants (and control i.e. unstressed plants), cDNA was prepared, and transcript analysis was performed using quantitative real-time (qRT-PCR) PCR (Table S1). The relative fold expression was calculated using the CT method; gene *β*-tubulin was used as an internal reference and compared with control plants^43^.

### Genome organisation and subcellular localisation

Plant (*S. brachiata*) genomic DNA was extracted, quantified using ND-1000 spectrophotometer and qualitatively analysed by agarose gel electrophoresis. The *SbSDR1* gene was amplified using genomic DNA as template with a gene-specific primer pair (Table S1), cloned and sequenced. The copy number of *SbSDR1* gene was determined using Southern blot analysis^6^,^26^. For subcellular localisation, a translational fusion cassette of *SbSDR1* along with RFP (red fluorescent protein) was generated using the gateway technology^25^,^26^. Expression cassette (RFP:*SbSDR1*) and control vector (pSITE-4CA:RFP) were transferred to onion epidermal cells using microprojectile bombardment (PDS-1000/He biolistic system, Biorad, USA) and transient expression of RFP was observed using an epifluorescence microscope (Axio Imager, Carl Zeiss AG, Germany).

### Heterologous expression and DNA binding assay

The coding sequence of the *SbSDR1* gene was cloned into the pET28a expression vector (Table S1), transformed into *E*. *coli* BL21 (DE3) cells and recombinant protein expression was induced^26^. The *SbSDR1* protein was purified, evaluated on SDS-PAGE (12%) and used for analysing DNA binding property by the electrophoretic mobility shift assay (EMSA). Genomic DNA, extracted from Salicornia and tobacco plants were digested (*Bam*H1), denatured at 94 °C for 5 min followed by cooling at 4 °C for 5 min and incubated with *SbSDR1* protein at 25 °C for 16 h. The *SbSDR1* protein and DNA/protein complexes were electrophoresed on non-denaturing polyacrylamide gel (6% native PAGE) at 50 V for three hours at 4 °C. The gel was silver stained, and electrophoretic mobility shift was studied.

### Genetic transformation of tobacco plants and molecular analysis

The *SbSDR1* CDS was amplified (Table S1), cloned in pCAMBIA2301 through an intermediate pRT101 plant expression vector and transformed into tobacco (*Nicotiana tabacum* cv. Petit Havana) plants by *Agrobacterium tumefacians* (strain LBA4404) mediated leaf disc method^44^. Putative transgenic tobacco plants were regenerated, and transgenic lines (T_0_ and T_1_) were screened by growing on kanamycin (50 mgL^−1^). The presence of transgenes was confirmed by PCR amplification (of *uid*A and *SbSDR1* genes), and integration (transgene events) was determined by Southern blot (Table S1). Transgenic lines (T_1_) were further screened for histochemical *β*-glucuronidase activity^45^ and lines showing high GUS activities were selected for further analysis. Overexpression of *SbSDR1* gene was analysed in selected transgenic lines by semiquantitative reverse transcriptase PCR (Rt-PCR), and the actin gene was used as an internal reference (Table S1).

### Abiotic stress treatments and evaluation of transgenic lines

All transgenic lines were maintained under controlled containment facility. Percent seed germination of transgenic plants was calculated under abiotic stress (salinity, 200 mM NaCl and osmotic, 300 mM mannitol) and compared with non-transgenic plants (wild type, WT) and unstressed (control) condition^46^. Seeds were germinated on MS containing kanamycin (50 mgL^−1^); germinated T_1_ transgenics and WT (germinated on only MS) seedlings were grown on MS supplemented with NaCl (200 mM) or mannitol (300 mM) for 21 days. Plant morphology, growth parameters, including shoot (SL) & root (RL) length, and fresh (Fw) & dry (Dw) weight were studied and compared with controls (WT and control condition; plants grown on MS only were taken as a control condition).

Plants (transgenic and WT) grown in control condition for 21 days were further transferred to hydroponics (½ MS) and plastic cups (containing garden soil) and grown for 30 days. Salinity (200 mM NaCl) or osmotic (300 mM mannitol) stress treatments were given for 24 hours to 30 days hydroponically grown plants. Physio-biochemical analyses, including electrolyte leakage, membrane stability index, relative water, proline, total soluble sugar, lipid peroxidation (MDA content), H_2_O_2_ content were performed for T_1_ transgenic lines under different abiotic stress treatments and compared with controls^47^,^48^,^49^,^50^,^51^,^52^,^53^. Similarly, generation of H_2_O_2_ and superoxide (O_2_^−^) was visualised by *in vivo* localisation studies^54^. Healthy leaves of 30 days grown (in hydroponics) plants (transgenic and WT) of a control condition were taken; 8 mm discs were punched out, subjected to stress condition (salinity, 200 mM NaCl or osmotic, 300 mM mannitol) and leaf senescence study was performed along with estimation of chlorophyll contents^21^,^23^,^55^,^56^.

Plants (transgenic and WT) transferred to plastic cups (containing garden soil) were grown for 30 days under green-house containment facility. For control condition, plants were irrigated (every alternate day) with ½ MS only, whereas irrigation with ½ MS containing 200 mM NaCl and no irrigation were considered salinity and drought stress treatment, respectively^5^. After 15 days of stress, plants were observed morphologically and compared with controls, whereas recovery study was performed for plants under drought stress by re-irrigating with tap water.

Plants (transgenic and WT) grown in plastic cups (for 30 days in green house under control condition) were shifted to controlled field condition and allow to grow further for 30 days. After that, salinity and drought stress were employed (as above) for 15 days and physiology including, net photosynthesis rate and stomatal conductance were measured by portable photosynthesis system (LI6400XT, LI-COR Biosciences, USA) and compared with controls.

### Expression profiling and microarray analysis

Abiotic stress (salinity, 200 mM NaCl or osmotic, 300 mM mannitol) was given to 30 days hydroponically grown plants (transgenic and WT) for 24 hours. Plants were harvested, total RNA was extracted for cDNA synthesis, and relative fold expression of selected transcription factors (*Nt*TFs) was studied by qRT-PCR. Extracted RNA of samples (WT and a selected transgenic line of control and treated with NaCl or mannitol) was converted to first strand cDNA. After that proceed to second strand cDNA synthesis, cRNA amplification, single stranded cDNA synthesis. Finally, fragmentation and terminal labelling were performed by following whole transcript (WT) expression arrays user manual (Affymetrix, USA). Labelled cDNAs were hybridised with tobacco whole transcript expression gene chip (containing total 272410 gene probes), washed and stained using fluidics module (GeneChip Fluidics Station 450, Affymetrix, USA) as per user instruction. Hybridised chips were scanned (Scanner 3000 7G, Affymetrix, USA), and scanned images were processed and analysed using expression console and transcriptome analysis console (Affymetrix, USA). Microarray analysis was performed in duplicate (n=2) and genes exhibiting significant fold expression (ANOVA *p*-value < 0.05) were considered for the study.

### Statistical analysis

Data from five replicates (for each set of the experiment), each containing fifteen plants were presented as mean ± SE and subjected to analysis of variance (ANOVA) to determine the significance of difference amongst the means of WT and transgenic plants of every treatment set. Data exhibited *p* < 0.05 was considered significantly different and designated by similar letters. All dataset was also analysed individually and in combination with principal component analysis (PCA), and respective heat maps were generated.

## Additional Information

**How to cite this article**: Singh, V. K. *et al*. A novel transcription factor-like gene SbSDR1 acts as a molecular switch and confers salt and osmotic endurance to transgenic tobacco. *Sci. Rep.* **6**, 31686; doi: 10.1038/srep31686 (2016).

## Supplementary Material

Supplementary Information

## Figures and Tables

**Table 1:** Selected transcripts that differentially expressed (up- or down-regulated) in *SbSDR1* overexpressing transgenic tobacco plant compared with the wild type under stress (salt or osmotic) conditions.

S. No.	Probe Id	Gene name	Gene accession	Fold Change (log2)
Transcripts significantly differentially expressed under salt (200 mM NaCl) stress				
Transporters				
1	NtPMIa1g79974e2_st	ABC transporter	FH327345	6.88
2	NtPMIa1g179413e1_s_st	ABC transporter 1 (NtPDR1 gene for PDR-type)	FH465185	5.91
3	NtPMIa1g58619e1_st	Sulphate transporter	FG143420	5.78
4	NtPMIa1g52967e1_st	Sugar transporter ERD6	ET768583	4.88
5	NtPMIa1g150213e1_s_st	ABC transporter G family member 11	FH150205	4.22
6	NtPMIa1g122574e1_st	ABC transporter B family member 21	FI074394	3.74
7	NtPMIa1g163746e1_st	ABC transporter C family member 10	FH377610	3.69
8	NtPMIa1g143376e1_st	ABC transporter C family member 3/4	FH290450	3.55
9	NtPMIa1g3211e1_st	Calcium-transporting ATPase	FI052025	3.45
10	NtPMIa1g61677e3_st	ABC transporter C family member 15	FH564659	3.34
11	NtPMIa1g11150e1_st	MRP-like ABC transporter	FH120467	3.29
12	NtPMIa1g168421e1_st	Sulphate transporter 3 (low affinity)	FH533814	3.15
13	NtPMIa1g129050e1_st	ABC transporter B family member 25/27	FH918583	3.07
14	NtPMIa1g9139e1_st	Anion transporter 2	ET929814	3.02
15	NtPMIa1g79753e1_st	Cation-chloride cotransporter 1	FH091636	−3.42
Stress proteins				
16	NtPMIa1g123767e1_st	DNA-binding protein SMUBP-2	FI077868	4.26
17	NtPMIa1g56604e1_s_st	NAC domain protein NAC5	ET045577	4.09
18	NtPMIa1g81083e1_st	REF/SRPP-like protein/or Stress-related protein	FG187326	3.66
19	NtPMIa1g44375e1_st	Calcium-binding protein/Calmodulin-like protein 4	EH617111	3.44
20	NtPMIa1g141096e1_st	Nuclear transport factor 2 (NTF2) family protein	FH231101	3.19
21	NtPMIa1g49504e2_st	Heat shock 70 kDa protein	ET710960	3.17
22	NtPMIa1g82819e3_st	ras-related protein Rab7-like/Small GTP-binding protein	ET802996	3.17
23	NtPMIa1g71923e1_s_st	G-box-binding factor 1-like	ET047835	3.16
Stress responsive genes/channels				
24	NtPMIa1g21515e1_s_st	PR-5 Gene	FH357120	6.91
25	NtPMIa1g138004e1_s_st	NtPOX1 (peroxidase)	FG146731	6.29
26	NtPMIa1g144683e1_st	str246C gene	FH548471	5.88
27	NtPMIa1g42792e1_st	Osmotin-like (OLP1)	FH990179	4.89
28	NtPMIa1g42154e2_st	Peroxidase	FH663398	4.79
29	NtPMIa1g29557e1_st	Sodium/hydrogen exchanger 8-like	ET764618	4.53
30	NtPMIa1g201506e1_st	Superoxide dismutase	EH619039	4.44
31	NtPMIa1g193487e1_st	Glutathione S-Transferase	ET691311	4.38
32	NtPMIa1g46700e1_st	Sodium/hydrogen exchanger	FH050971	4.04
33	NtPMIa1g4232e2_s_st	Aquaporin TIP3-2 (tonoplast intrinsic protein)	FH043168	3.83
34	NtPMIa1g35785e3_st	Potassium channel AKT1-like	ET966045	3.70
35	NtPMIa1g46700e3_s_st	Na+/H+ antiporter/or sodium/hydrogen exchanger	FI072621	3.55
36	NtPMIa1g47884e3_st	Calcium channel (two pore) protein 1	FH134749	3.46
Transcription factors				
37	NtPMIa1g31907e2_s_st	Transcription factor (WRKY) 31	FG161188	5.64
38	NtPMIa1g80200e5_st	Transcription factor TGA1/Basic leucine zipper protein	FG197996	4.60
39	NtPMIa1g214476e1_st	Transcription factor (WRKY) 6	ET043797	4.17
40	NtPMIa1g51042e1_st	Transcription factor ILR3-like	ET854451	3.94
41	NtPMIa1g50790e2_st	Transcription factor TCP3	ET046638	3.88
42	NtPMIa1g43220e3_st	Transcription factor (WRKY) 21	FH187095	3.72
43	NtPMIa1g155166e1_st	Transcription factor LUX	ET694713	3.62
44	NtPMIa1g50790e1_st	Transcription factor TCP4-like	ET980639	3.57
45	NtPMIa1g53553e2_x_st	Transcription factor Bhlh041	FH670186	3.53
46	NtPMIa1g32403e1_st	Transcription factor (WRKY) 43	ET049847	3.40
47	NtPMIa1g99480e1_s_st	Transcription factor (GATA) 25	FH991549	3.17
48	NtPMIa1g80077e2_st	transcription factor JUNGBRUNNEN 1-like	FI039736	3.03
49	NtPMIa1g178470e1_s_st	Transcription factor family-PLATZ- protein	ET042575	−3.15
50	NtPMIa1g93305e1_st	Ethylene-responsive transcription factor	FH965889	−3.17
Uncharacterised				
51	NtPMIa1g184611e1_s_st	Uncharacterised	EH617968	6.21
52	NtPMIa1g112984e1_s_st	Uncharacterised	ET991956	5.60
53	NtPMIa1g59636e1_st	Uncharacterised	FH622649	5.58
54	NtPMIa1g127287e1_st	Uncharacterised	FH333853	−5.69
55	NtPMIa1g93939e1_st	Uncharacterised	FH331817	−3.38
56	NtPMIa1g125877e1_x_st	Uncharacterised	FI084463	−3.29
Zinc finger protein				
57	NtPMIa1g1891e1_s_st	Zinc induced facilitator like	FI027112	5.19
58	NtPMIa1g97020e1_st	Zinc metalloprotease (ATP-dependent)	FH980189	3.78
Transcripts significantly differentially expressed under osmotic (300 mM mannitol) stress				
Transporters				
1	NtPMIa1g179293e1_st	ABC transporter G family member 35	FH628071	4.01
2	NtPMIa1g200291e1_st	ABC transporter B family member 21	FH968229	3.35
3	NtPMIa1g118531e1_st	ABC transporter A family member 11	ET925380	2.88
4	NtPMIa1g19057e2_st	ABC transporter G family member 3	FH326473	2.85
5	NtPMIa1g173628e1_s_st	Sucrose transport protein SUC3	ET942104	2.71
6	NtPMIa1g42126e1_s_st	ABC transporter B family member 25	FH321728	2.55
7	NtPMIa1g47623e1_st	Sulfate transporter	FH062478	−3.64
Stress responsive proteins/genes/channels				
8	NtPMIa1g136207e1_st	Protein kinase (receptor-like)	FH084028	4.92
9	NtPMIa1g78543e3_st	Aquaporin-4	FH137367	2.76
10	NtPMIa1g57744e1_st	Anionic peroxidase gene/Peroxidase	FH308715	2.68
11	NtPMIa1g82649e2_st	Plasma membrane H+ ATPase	FH091813	2.43
12	NtPMIa1g190637e1_x_st	Mitogen-activated protein kinase kinase kinase NPK1	FH176408	2.41
13	NtPMIa1g17460e1_s_st	Heat shock protein (class II)	FH127057	−3.54
14	NtPMIa1g40121e1_st	Cytochrome P450	FG196194	−3.09
15	NtPMIa1g201049e1_st	Heat shock protein 101	ET915891	−2.98
Transcription factors				
16	NtPMIa1g2222e1_st	Transcription factor (WRKY) 41	FI020184	2.87
17	NtPMIa1g82333e1_st	Transcription factor bHLH55	FH551099	2.38
18	NtPMIa1g122488e1_st	Transcription factor (heat stress) B-2a	ET044925	−2.52
19	NtPMIa1g123702e2_st	Transcription factor (WRKY) 33	FI077699	−2.33
Uncharacterised				
20	NtPMIa1g140270e1_st	Uncharacterised	FG168624	3.49
21	NtPMIa1g82079e2_st	Uncharacterised	ET983581	3.35
22	NtPMIa1g38269e1_st	Uncharacterised	ET920361	−3.26
23	NtPMIa1g180425e1_s_st	Uncharacterised GPI-anchored protein	FH467984	−2.96

No sign indicates up-regulation, whereas “−” sign shows down-regulation. Fold-expression is significant at ANOVA *p* < 0.05.
