# Corrigendum: A novel transcription factor-like gene *SbSDR1* acts as a molecular switch and confers salt and osmotic endurance to transgenic tobacco

**DOI:** 10.1038/srep35128

**Published:** 2016-11-10

**Authors:** Vijay Kumar Singh, Avinash Mishra, Intesaful Haque, Bhavanath Jha

Scientific Reports 6: Article number: 3168610.1038/srep31686; published online: 08 23 2016; updated: 11 10 2016.

This Article contains typographical errors. In the legend of Figure 6, the descriptions corresponding to Figures 6c and 6d have been mislabelled ‘(D)’ and ‘(E)’ respectively. In the Results section,

“In total, 1,998 genes were differentially expressed by more than eight-fold (<−8 or >8) under salinity stress,”

should read:

“In total, 1,198 genes were differentially expressed by more than eight-fold (<−8 or >8) under salinity stress,”

