# Author Correction: A novel transcription factor-like gene *SbSDR1* acts as a molecular switch and confers salt and osmotic endurance to transgenic tobacco

**DOI:** 10.1038/s41598-020-62996-8

**Published:** 2020-04-10

**Authors:** Vijay Kumar Singh, Avinash Mishra, Intesaful Haque, Bhavanath Jha

**Affiliations:** Division of Marine Biotechnology and Ecology, CSIR-Central Salt and Marine Chemicals Research Institute, G. B. Marg, Bhavnagar, (Gujarat) India

Correction to: *Scientific Reports* 10.1038/srep31686, published online 23 August 2016

This Article contains an error in the Methods section, under subheading ‘Abiotic stress treatments and evaluation of transgenic lines’, where:

“Plants (transgenic and WT) grown in plastic cups (for 30 days in green house under control condition) were shifted to controlled field condition and allow to grow further for 30 days.”

should read:

“Plants (transgenic and WT) grown in plastic cups (for 30 days in green house under control condition) were shifted to controlled field condition (in green house; not open field) and allowed to grow further for 30 days.”

