# Supplementary material for: A novel transcription factor-like gene SbSDR1 acts as a molecular switch and confers salt and osmotic endurance to transgenic tobacco
Source: Sci Rep. 2016 Aug 23;6:31686. doi: 10.1038/srep31686 (PMC4994045; doi:10.1038/srep31686)
Supplement: Supplementary Information [file srep31686-s1.pdf]

Supplementary data

A novel transcription factor-like gene *SbSDR1* acts as a molecular switch and confers salt and osmotic endurance to transgenic tobacco

Vijay Kumar Singh, Avinash Mishra*, Intesaful Haque and Bhavanath Jha*

Division of Marine Biotechnology and Ecology, CSIR-Central Salt and Marine Chemicals Research Institute, G. B. Marg, Bhavnagar (Gujarat), India

*Corresponding authors

Tel: +91-278-2567760 Ext. 6260; Fax: +91-278-2567562; e-mails: avinash@csmcri.org (AM) and bjha@csmcri.org (BJ)

Table S1: Primers used in the study and PCR conditions

Purpose	Primer	Orientation	Sequence (5'-3')	PCR conditions
RACE	GSP5'R1	Reverse	GCCTTTAGCCATACCCATCAA	As suggested 5' RACE System for Rapid Amplification of cDNA Ends, version 2.0
	GSP5'R2	Reverse	CGGCTCTTTCTCCTTGTCTTTA	
	GSP5'R3	Reverse	CCTTCAGCCTTGGTTACATACTG	
Full length gene, Genomic organization, semi quantitative Rt PCR and confirmation of transgenic lines	SDR1-F	Forward	ATGGAAGGAGAAGCAGC	[94°C-5min] x1; [94°C-30sec, 55°C-30sec, 72°C-30sec] x30; [72°C-10min] x1
	SDR1-R	Reverse	TCACTTATTAAAGAGGCCTT	
Transcript profiling	SDR1RT-F	Forward	CAAAAACAGAGCATGGAGCA	[94°C-5min] x1; [94°C-10sec, 60°C-30sec] x40 followed by melt curve analysis
	SDR1RT-R	Reverse	CTGTTTTCGTCGAGCTTTCC	
Cloning into pET28a expression vector	SDR1pET-F	Forward	CCCAAGCTTATGGAAGGAGAAGCAGC	[94°C-5min] x1; [94°C-30sec, 58°C-30sec, 72°C-30sec] x30; [72°C-10min] x1
	SDR1pET-R	Reverse	CCGCTCGAGTCACTTATTAAAGAGGCCTT	
Probe for Southern hybridization (copy number)	SDR1RT-F	Forward	CAAAAACAGAGCATGGAGCA	[94°C-5min] x1; [94°C-30sec, 55°C-30sec, 72°C-30sec] x30; [68°C-7min] x1
	SDR1RT-R	Reverse	CTGTTTTCGTCGAGCTTTCC	
For expression of transcription factor and other genes	NtAP2-F	Forward	AAGGGCGAGGAAGAACAAAT	[94°C-5min] x1; [94°C-10sec, 60°C-30sec] x40 followed by melt curve analysis
	NtAP2-R	Reverse	GTGGCTCTGGAAAGTTGA	
	NtPLC-F	Forward	CTGATGACGGAGCAGGTGT	
	NtPLC-R	Reverse	CTGAGCAAGTTCCGCTGAG	
	NtLEA5-F	Forward	CATCTCTGCTTTCGTCGTTG	
	NtLEA5-R	Reverse	AGCATGACTCTCTGGCCTGT	
	NtP5CS-F	Forward	CTCCCTGCTCAAGATTCCA	
	NtP5CS-R	Reverse	ACAGCAGCACTGTCAACCTG	
	NtERF8-F	Forward	TCCAAGCCCAAATCAACTTC	
	NtERF8-R	Reverse	GGGCAATCGGATCTCAACTA	
Subcellular localization	SDR1CA-F	Forward	CACCATGGAAGGAGAAGCAGC	[94°C-5min] x1; [94°C-30sec, 55°C-30sec, 72°C-30sec] x35; [72°C-10min] x1
	SDR1CA-R	Reverse	TCACTTATTAAAGAGGCCTT	
Cloning in pRT101	SDR1pRT-F	Forward	TCCGAGCTCATGGAAGGAGAAGCAGC	[94°C-5min] x1; [94°C-30sec, 58°C-30sec, 72°C-30sec] x30; [72°C-10min] x1
	SDR1pRT-R	Reverse	CGCGGATCCTCACTTATTAAAGAGGCCTT	
Reference genes	Sbβ-tubline-F	Forward	GGAGTCACCGAGGCAGAG	
	Sbβ-tubline-R	Reverse	ATCACATATCAGAAACCACAA	
	uidA-F	Forward	GATCGCGAAAACTGTGGAAT	
	uidA-R	Reverse	TGAGCGTCGCAGCAGAACATTAC	
	NtActin-F	Forward	CGTTTGGATCTTGCTGGTCGT	
	NtActin-R	Reverse	CAGCAATGCCAGGGAACATAG	

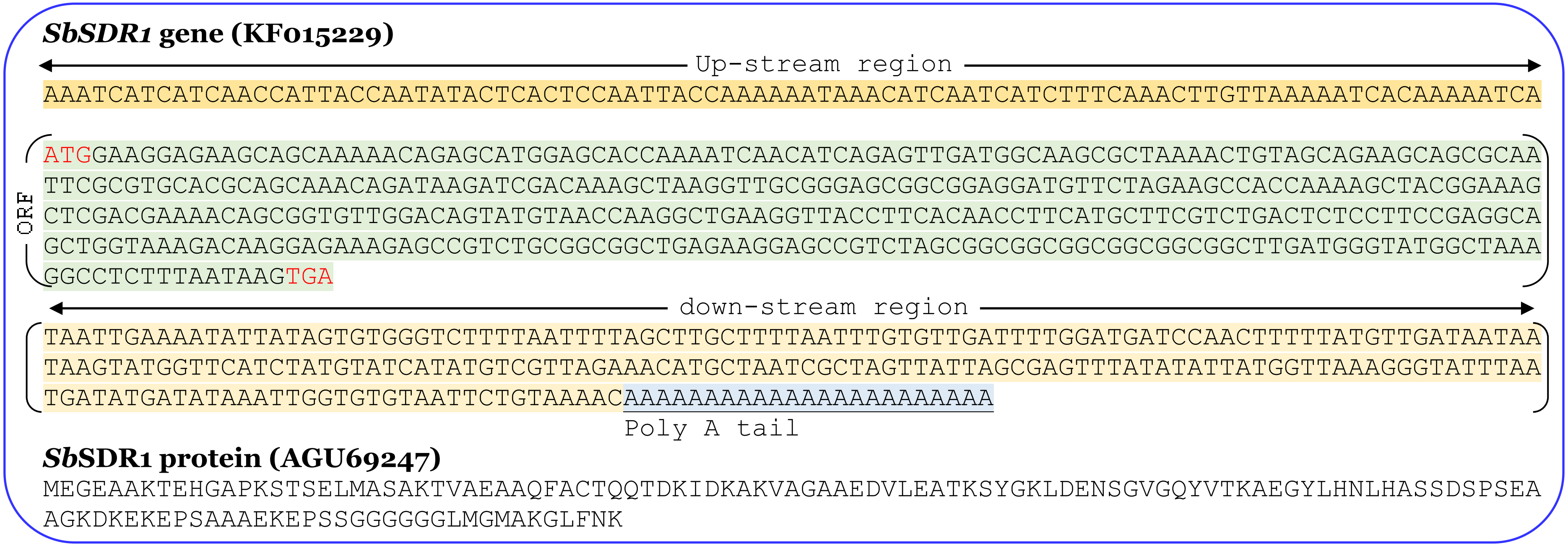

Figure S1: Graphical representation of *SbSDR1* gene. The *SbSDR1* cDNA sequence was 728 bp long and consisted of a 5′-untranslated leader sequence (5′-UTR; 1–93 bp), an open reading frame (ORF; 94–483 bp), a 3′-UTR (483–705 bp) and a poly(A) tail of 23 base pairs. The 390-bp ORF encodes a peptide of 129 amino acids.

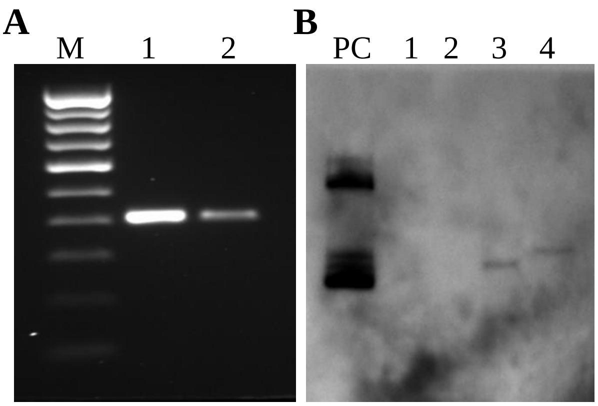

Figure S2: Genome organization. (A) PCR amplification of *SbSDR1* gene from genomic DNA and cDNA; and (B) Southern hybridisation of *S. brachiata* genome to determine copy number of *SbSDR1* gene.

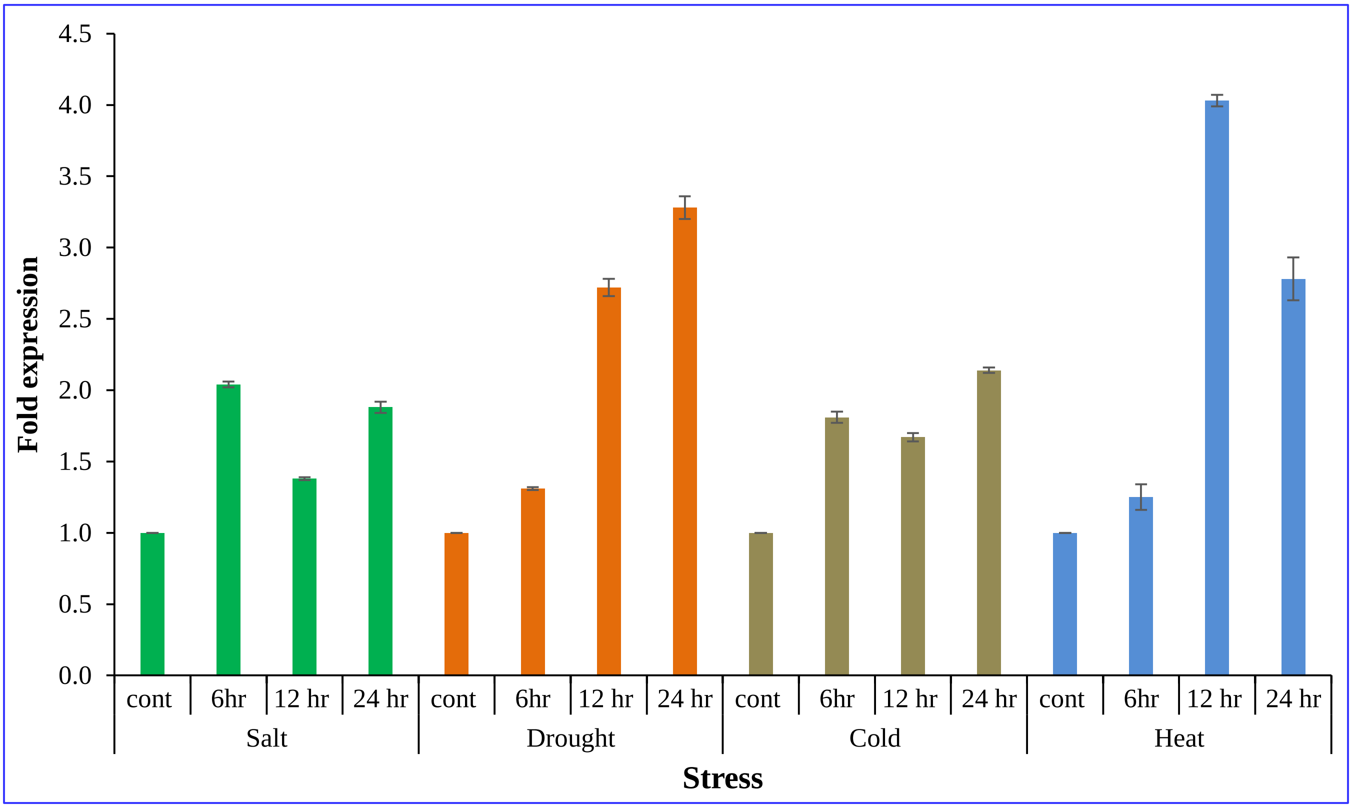

Figure S3: Transcription profiling of the *SbSDR1* gene under different abiotic stress condition.

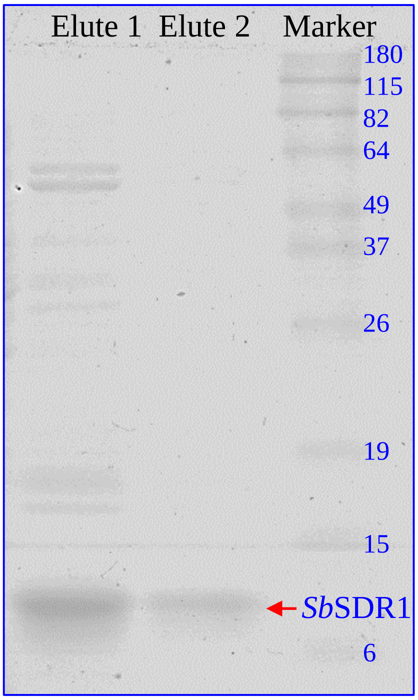

Figure S4: SDS-PAGE of the *Sb*SDR1 recombinant protein expressed in *E.coli*.

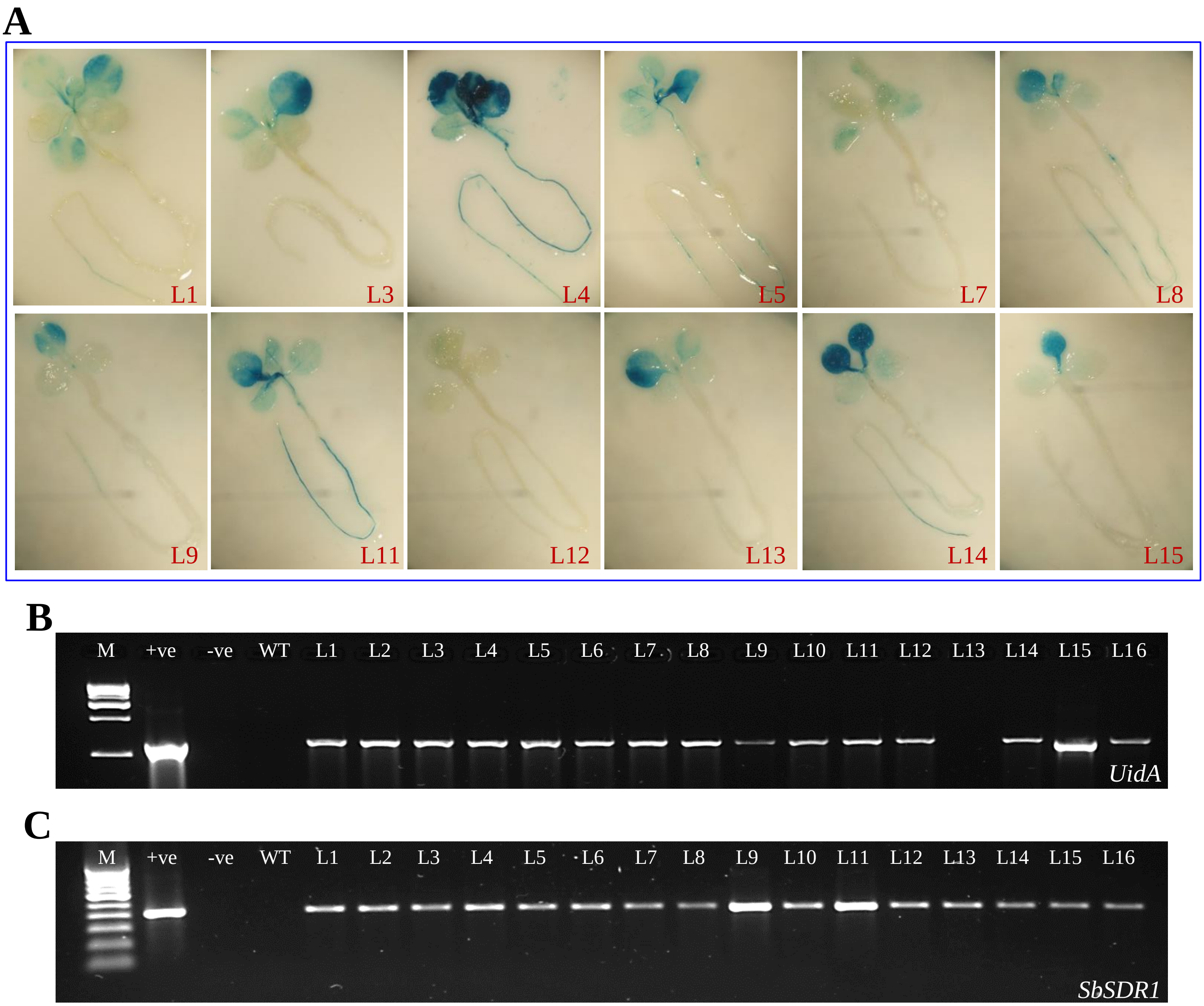

Figure S5: Confirmation of putative transgenic lines. (A) Histochemical GUS assay, amplification of (B) *UidA* gene and (C) *SbSDR1* gene in different transgenic lines. M: molecular marker, +ve: positive control, -Ve: negative control, WT: wild type plant (non-transformed) and L1-L16: transgenic tobacco lines.

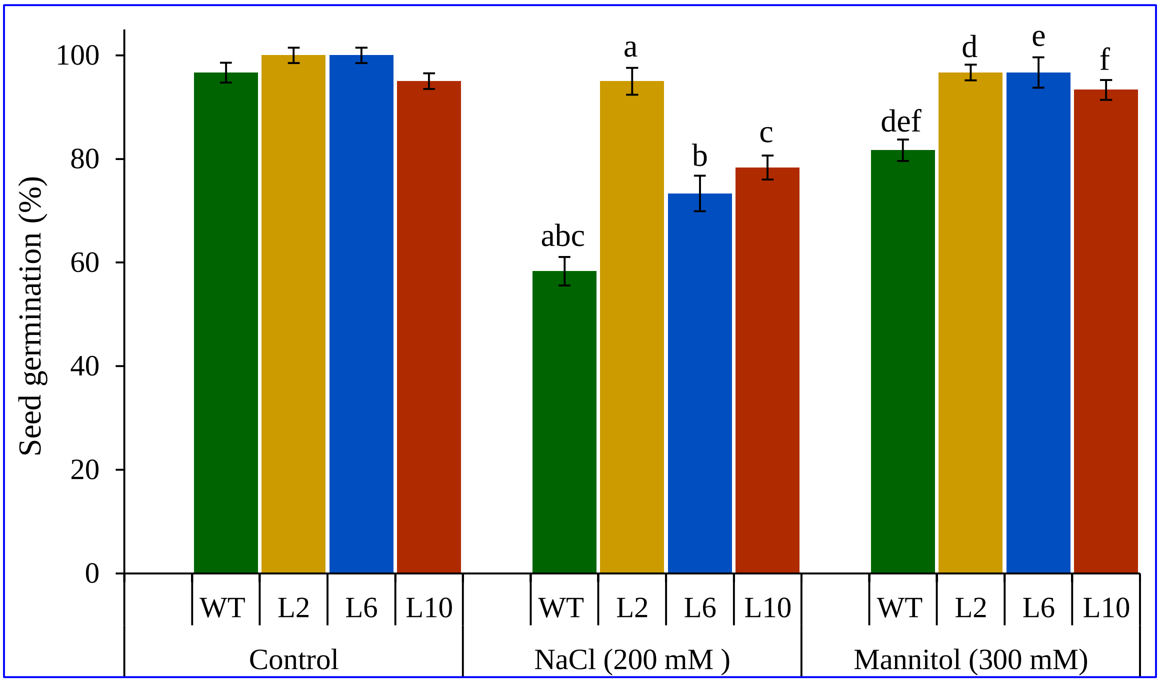

Figure S6: Percent seed germination of transgenic tobacco plants under abiotic stress. WT and transgenic lines (L2, L6 and L10) were germinated under salinity and osmotic stress condition. Bars represent means ± SE and values with similar letters are significant at $P < 0.05$.

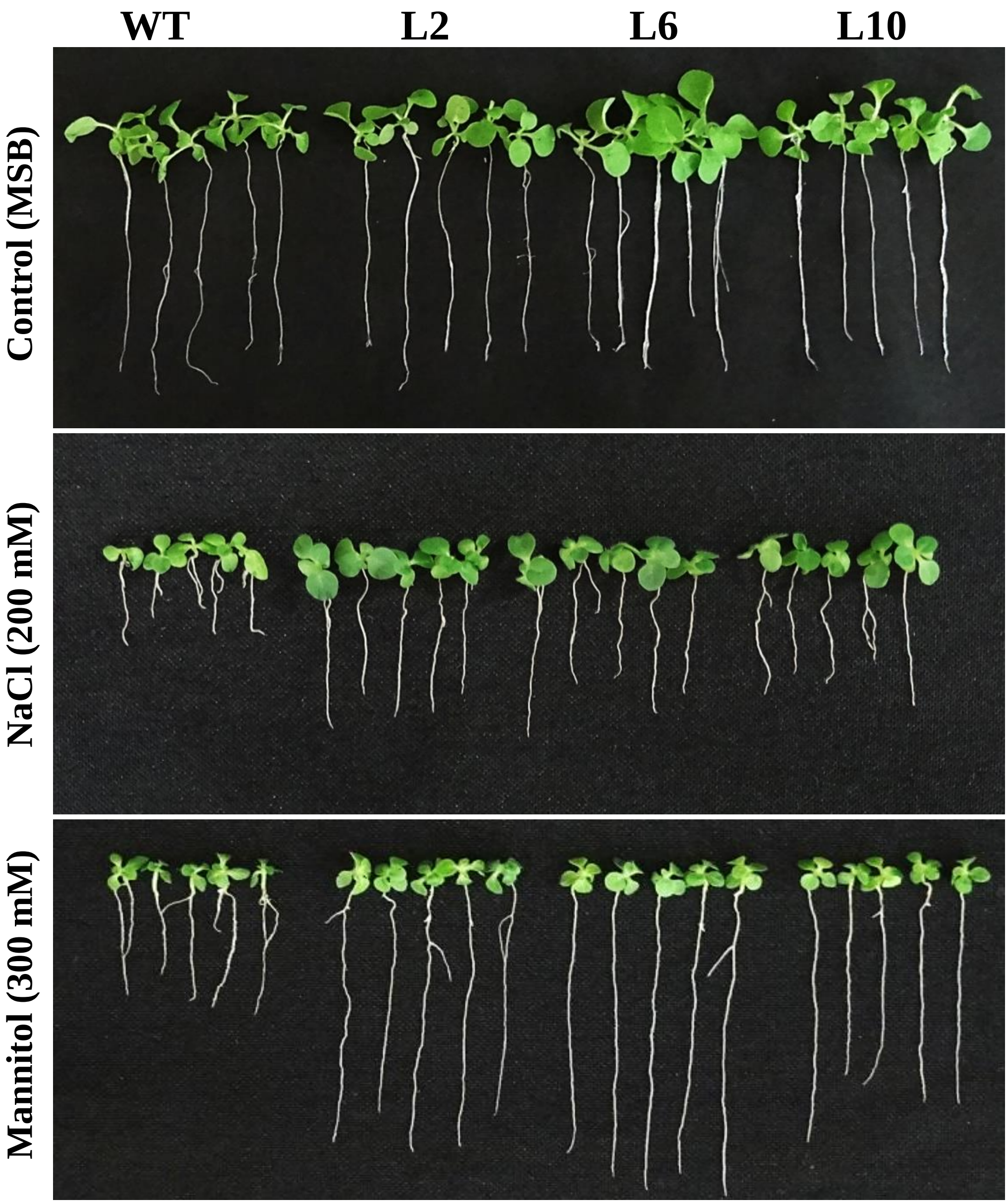

Figure S7: Comparative growth analysis of WT and transgenic lines under control and stress conditions.

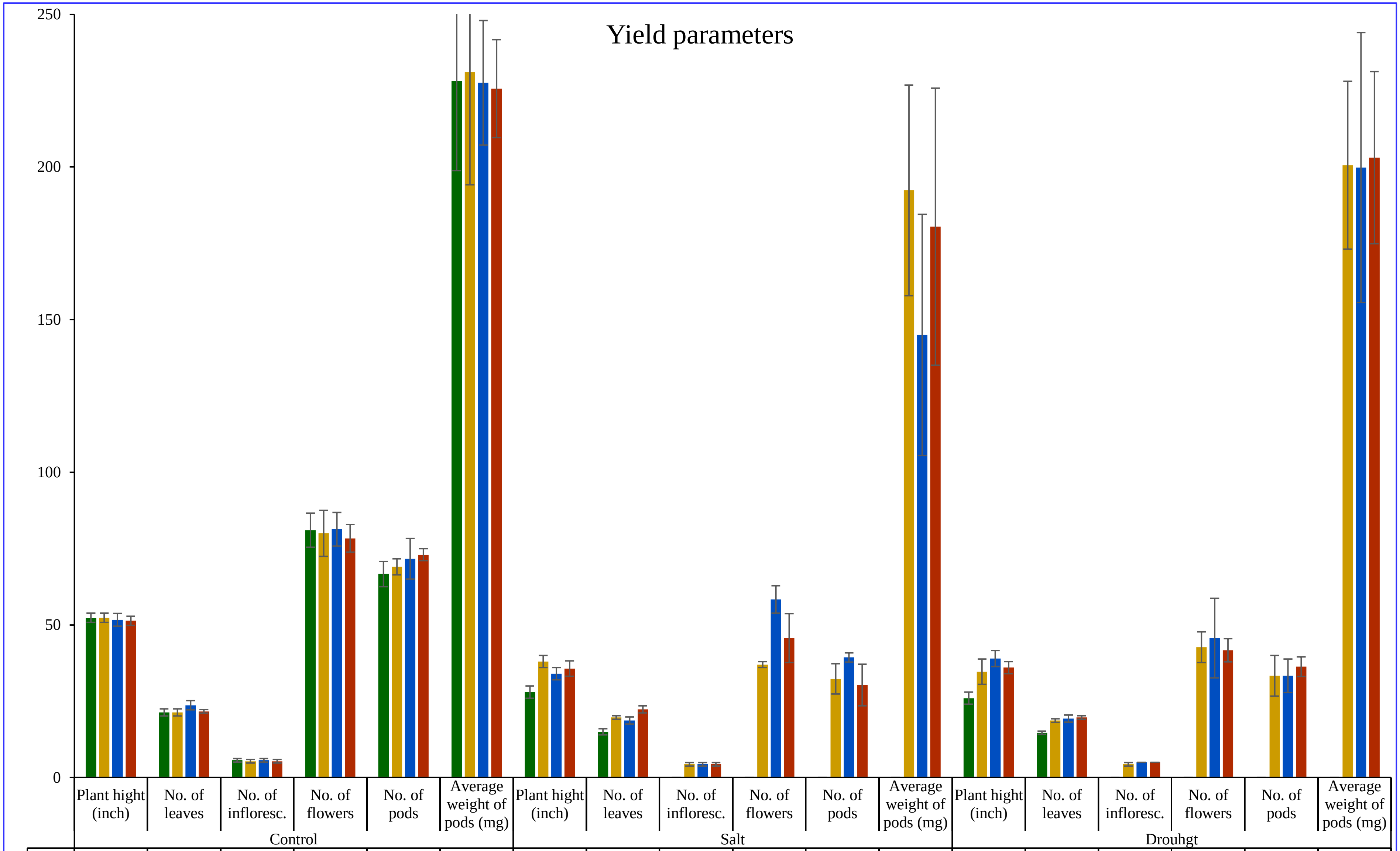

	Control: Plant hight (inch)	Control: No. of leaves	Control: No. of infloresc.	Control: No. of flowers	Control: No. of pods	Control: Average weight of pods (mg)	Salt: Plant hight (inch)	Salt: No. of leaves	Salt: No. of infloresc.	Salt: No. of flowers	Salt: No. of pods	Salt: Average weight of pods (mg)	Drouhgt: Plant hight (inch)	Drouhgt: No. of leaves	Drouhgt: No. of infloresc.	Drouhgt: No. of flowers	Drouhgt: No. of pods	Drouhgt: Average weight of pods (mg)
WT	52.33	21	6	81	67	228.16	28.00	15	0	0	0	0	26.00	15	0	0	0	0
L2	52.33	21	5	80	69	231.10	38.00	20	4	37	32	192.36	34.67	19	4	43	33	200.58
L6	51.67	24	6	81	72	227.58	34.00	19	4	58	39	144.95	39.00	19	5	46	33	199.78
L10	51.33	22	5	78	73	225.67	35.67	22	4	46	30	180.47	36.00	20	5	42	36	203.02

Figure S8: Yield parameters of WT and *SbSDR1* overexpressing transgenic lines under non-stress and stress conditions. All data were recorded as per stages of plants grown under control (non-stressed) condition.

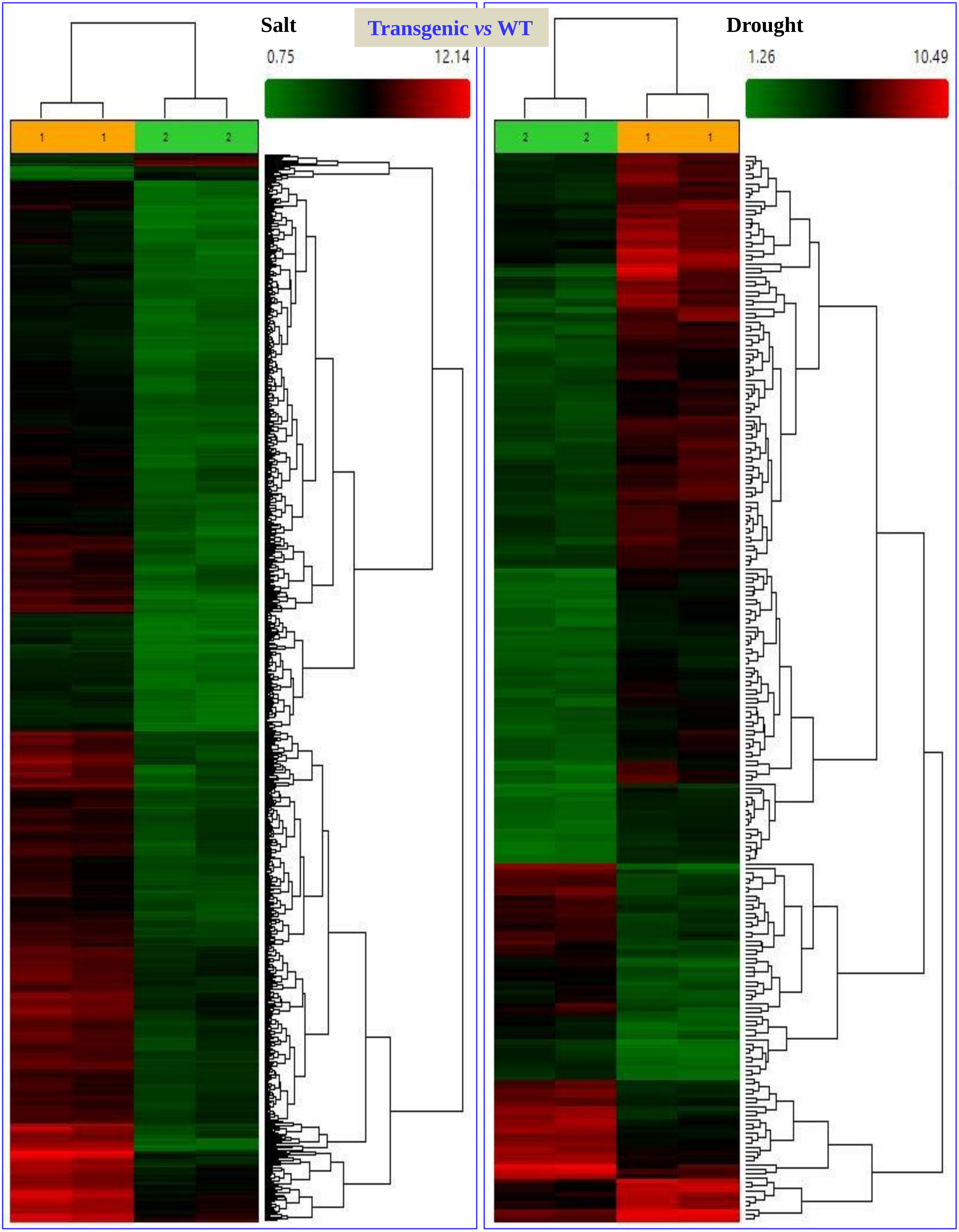

Figure S9: Hierarchical cluster analysis of *SbSDR1* transgenic tobacco plant showing differentially expressed genes under salt and osmotic (drought) stress conditions compared to WT plant.

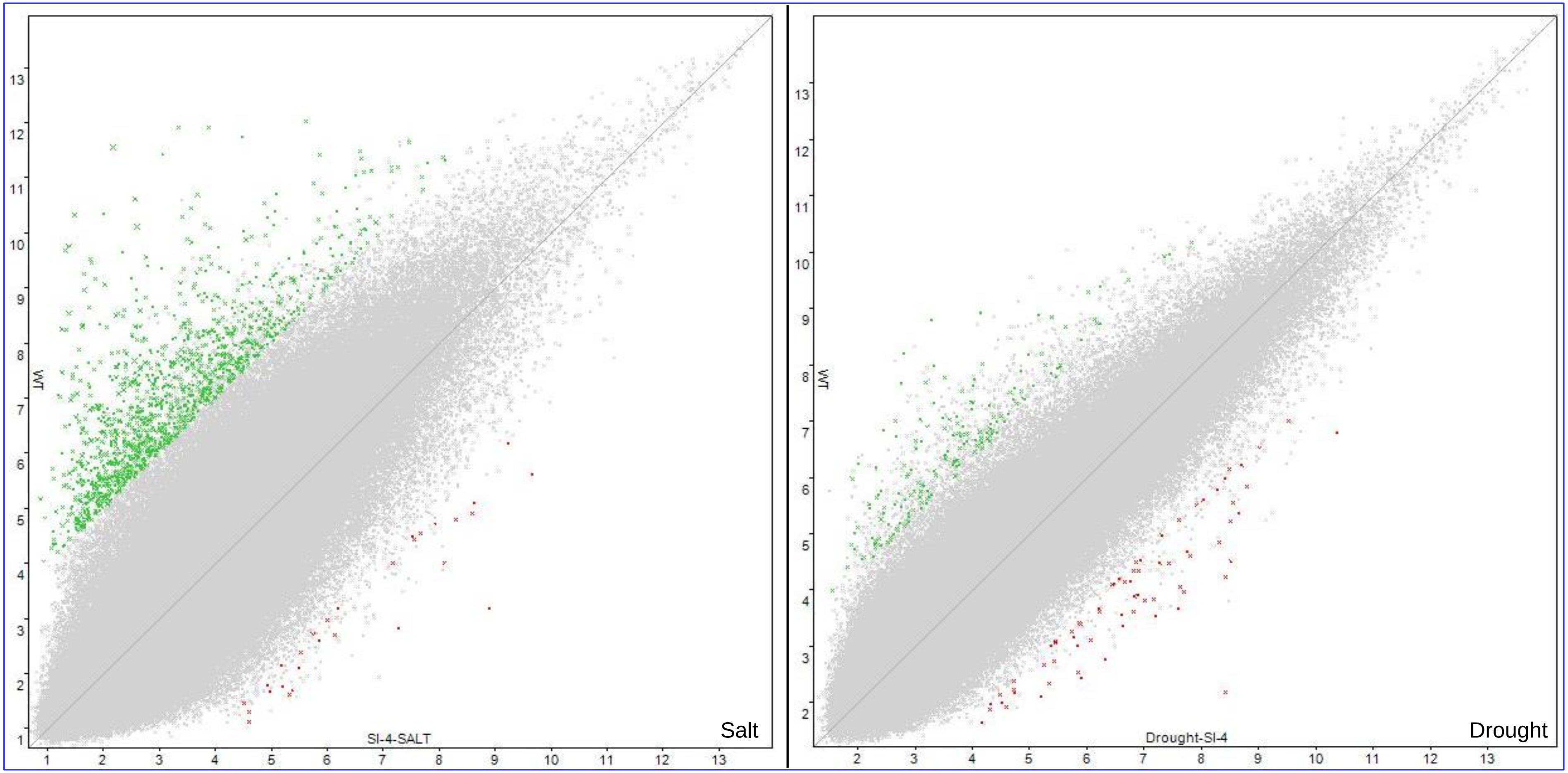

Figure S10: Scattered plot obtained from microarray analysis of *SbSDR1* transgenic tobacco plant showing differentially expressed genes under salt and osmotic (drought) stress conditions compared to WT plant.

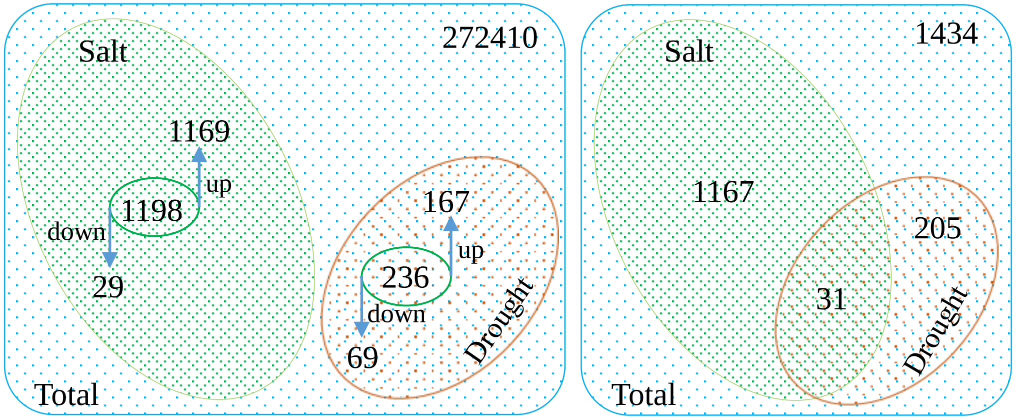

Figure S11: Venn diagram showing genes differentially expressed in *SbSDR1* transgenic plant compared to WT under salt and drought stress as studied by microarray.

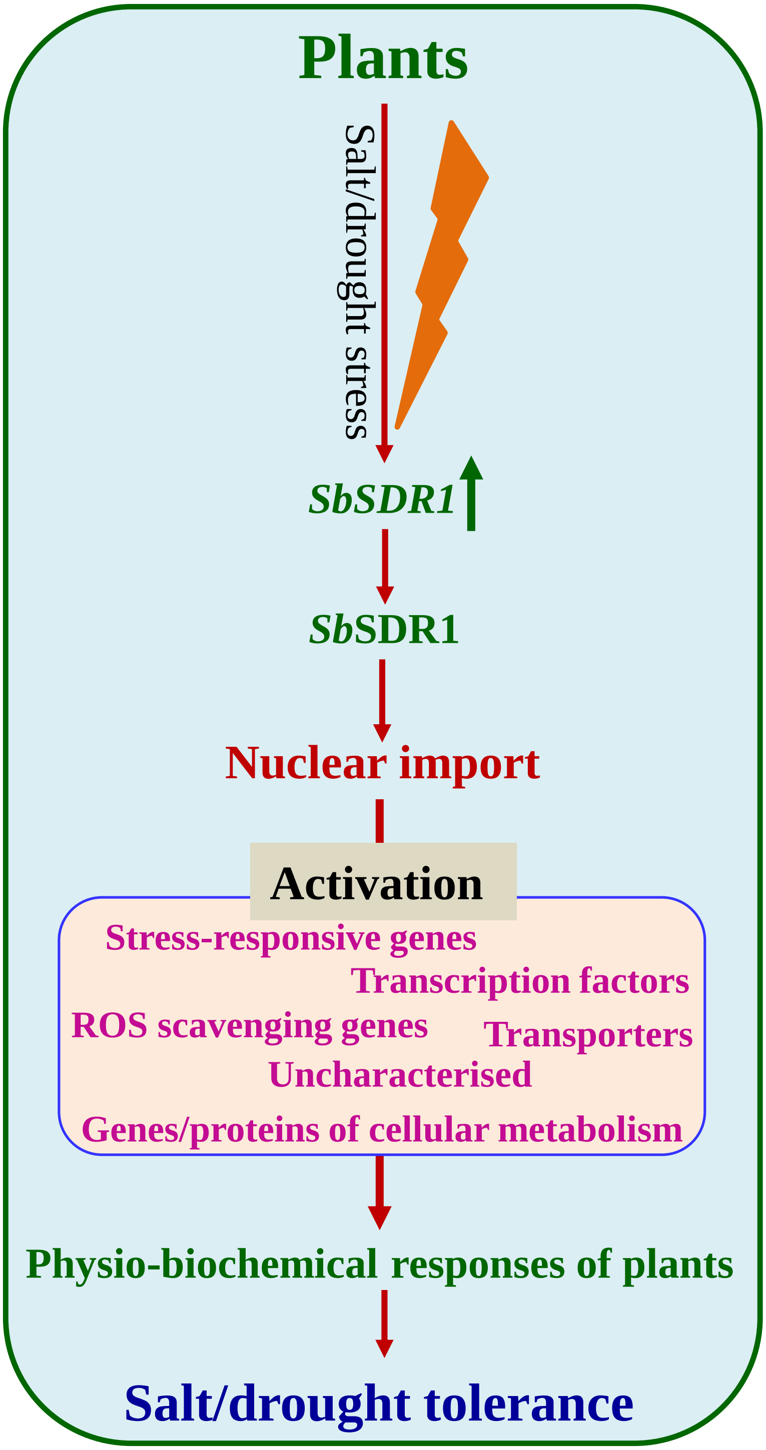

Figure S12: A hypothetical model explaining probable role of the *SbSDR1* in abiotic stress tolerance system.
